# Lessons and perspectives for applications of stochastic models in biological and cancer research

**DOI:** 10.6061/clinics/2018/e536s

**Published:** 2018-09-11

**Authors:** Alan U Sabino, Miguel FS Vasconcelos, Misaki Yamada Sittoni, Willian W Lautenschlager, Alexandre S Queiroga, Mauro CC Morais, Alexandre F Ramos

**Affiliations:** I Escola de Artes Ciências e Humanidades (EACH), Universidade de Sao Paulo, Sao Paulo, SP, BR; II Departamento de Radiologia e Oncologia, Instituto do Cancer do Estado de Sao Paulo (ICESP), Faculdade de Medicina FMUSP, Universidade de Sao Paulo, Sao Paulo, SP, BR

**Keywords:** Markov Chains, Models, Theoretical, Stochastic Processes, Regulation, Gene Expression, Contact Inhibition

## Abstract

The effects of randomness, an unavoidable feature of intracellular environments, are observed at higher hierarchical levels of living matter organization, such as cells, tissues, and organisms. Additionally, the many compounds interacting as a well-orchestrated network of reactions increase the difficulties of assessing these systems using only experiments. This limitation indicates that elucidation of the dynamics of biological systems is a complex task that will benefit from the establishment of principles to help describe, categorize, and predict the behavior of these systems. The theoretical machinery already available, or ones to be discovered to help solve biological problems, might play an important role in these processes. Here, we demonstrate the application of theoretical tools by discussing some biological problems that we have approached mathematically: fluctuations in gene expression and cell proliferation in the context of loss of contact inhibition. We discuss the methods that have been employed to provide the reader with a biologically motivated phenomenological perspective of the use of theoretical methods. Finally, we end this review with a discussion of new research perspectives motivated by our results.

## INTRODUCTION

Description of physical phenomena by theoretical methods has motivated the construction of a rich machinery ranging across general relativity (description of the behavior of matter at the macroscopic scale), quantum mechanics (description of the behavior of matter at the microscopic scale), electro-magnetism (description of electric charges, magnetic dipoles and light-related phenomena), and condensed matter theory (microscopic description of solid-state systems). These tools have enabled the control and design of specific experiments which outcomes are predicted within specific error ranges, as well the development of new technologies derived from the knowledge that those tools motivated. Fortunately, theories have a scope of applicability, i.e., they do not explain all observed data related to a given phenomenon. In general, this limitation has led to the development of new theories that may lead to additional verifiable hypothesis. For example, in contrast to Newtonian gravity, general relativity successfully predicts precession of the perihelion of Mercury or light bending by the sun. Furthermore, experiments aimed at investigating different manifestations of a phenomenon would require the development of specific theoretical or technological tools. For example, one may consider the use of tensor calculus in general relativity instead of vector calculus of Newtonian gravitation or the high-precision instruments required for detection of gravitational waves. Biology, however, has followed a different historical trajectory, with the predominant use of experimental methods. Biologists also rely on qualitative models to help construct a static picture of biological phenomena. This approach has relevant scientific and technological implications. Examples include the establishment of evolutionary theory—a key paradigm of modern science—or the ability to control biological phenomena at the molecular level, as occurs in the production of human insulin. However, this strategy has a clear limit if one is interested in the dynamics resulting from the interactions of many compounds at different levels of living matter, such as organisms, tissues, cells and molecules. Additionally, interactions among components at different levels give rise to a highly complex picture whose description will demand the use of all machinery available in the scientific toolbox. These techniques include the use of mathematical methods not only as a number-crunching technique but also as a strategy for formulating new principles to describe biological phenomena, for testing hypotheses that cannot be assessed experimentally, and, in the case of successful theories, for predicting the outcomes of different experimental designs or guiding the development of new technologies.

In this mini-review, we explain the usefulness of quantitative techniques in the investigation of biological phenomena. We consider the application of stochastic methods to describe phenomena occurring at the molecular and cellular levels. The first topic will be reviewed within the scope of a two-state stochastic model to quantify the expression of a gene that is either self-repressed or externally regulated. We approach the second topic with a stochastic model to quantify the role of contact inhibition in a co-culture *in vitro* experiment combining keratinocytes and melanoma cells. Our investigations have enabled us to understand how the synthesis of gene products is influenced by the promoter's ON and OFF switching at the molecular level. More specifically, we have shown that self-repression is the only mechanism required for noise reduction in protein numbers 1. Furthermore, we used this model to approach noise in the development of *Drosophila melanogaster* embryos to understand how an externally regulated gene produces mRNA during development with a proper spatio-temporal pattern 2. At the cellular level, the mechanism for cell proliferation control called contact inhibition was quantified as an exclusion diameter between cells. This mechanism enables the formation of clusters of melanoma cells in co-culture with keratinocytes 3. Furthermore, the model predicts that melanoma cells will prevail in a given spatial domain, if one observes the cell population dynamics during a sufficiently long period, because of their low degree of contact inhibition (or smaller exclusion diameters).

The intrinsic randomness of biological phenomena justifies the use of a stochastic approach to investigate these processes. At the intracellular level, randomness is caused by low copy numbers of chemical reactants and their heterogeneous distribution inside the cell 4. For example, random fluctuations have been widely observed in gene expression of both prokaryotic and eukaryotic cells by fluorescence techniques 5-20. Interestingly, however, the noise shape may be controlled by different gene regulatory strategies, such as self-repression that leads to low noise regimes 1,. Alternatively, external regulation has been identified as a gene regulatory strategy that results in increased noise 5,25,26. These results suggest self-repression as a unique mechanism controlling gene expression when high precision is necessary, as is the case during development. However, recent results have shown that external regulation may be sufficient to generate the required spatial precision for the formation of stripes of gene expression along the anterior-posterior (AP) axis of a *D. melanogaster* embryo 2,27,28.

Indeed, developmental processes require high precision to control the production of specific gene products to ensure that they are present at the proper locations and times during the life of an organism. This fact may lead to the perception that noise is always detrimental to the cell. Such a premise is not always true. For example, individual cells increase their chance of survival under stress conditions via noise in gene expression and the consequential generation of phenotypic diversification 29-32. However, normally behaving tissues are characterized by well-organized cellular structures along space and time. This organization is achieved by homeostatic mechanisms controlling cell densities in tissues. However, molecular fluctuations may affect cell genetics, modify the regulation of proliferation-related gene expression to favor cell duplication, and induce the appearance of carcinoma *in situ*. The latter generates spatially disorganized cell structures in tissues, disrupts homeostasis, and provides conditions for an invasive cell phenotype to appear. Thus, a manifestation of stochasticity is a beneficial trait for cancer cells (at the individual level), but at the organism level, the noise eventually has lethal effects.

Therefore, an important challenge in cancer biology is to determine the mechanisms underlying the progression of a carcinoma *in situ* and how these cells become prevalent within a region for a sufficiently long interval such that an invasive phenotype appears. One important mechanism necessary for the prevalence of tumor cells is the loss of contact inhibition 33,34. Contact inhibition of proliferation in culture experiments is associated with the ability of cells to maintain their density in a given tissue at optimal values 35,36. Loss of this ability causes cancer cells to keep proliferating in culture experiments even when confluence is reached 33. In contrast, it has been shown that hypersensitivity to contact inhibition in fibroblasts of naked mole rats is a mechanism that stops proliferation at low cell densities in culture experiments, which is caused by the interplay between the p16 and p27 cyclin-dependent kinase inhibitors. When these proteins are expressed together, both can inhibit proliferation at lower cell densities than that when p16 is not expressed 37. These experimental results suggest the necessity of a quantitative description of the intensity of contact inhibition in normal or cancer cells to enhance our ability to predict or describe carcinoma *in situ* growth.

The next sections are devoted to an overview of the three applications mentioned above and to some research perspectives. We start with the stochastic model for regulation of gene expression. We explain the chemical kinetics that enables the self-repressing gene to be expressed at low noise regimes. Furthermore, we present our results in the context of development of *D. melanogaster* embryos, which indicates the possibility of using this model to approach complex organisms. Then, we move on to the cell level approach to quantify the degree of contact inhibition between two cells as an exclusion diameter. Lower degrees of contact inhibition are indicated by smaller exclusion diameters, and this method is applied to describe a co-culture experiment with melanoma cells and keratinocytes. We present some possibilities for future investigations in the last section.

### Random fluctuations in gene expression

Randomness in gene expression has been measured in terms of the Fano factor, defined as the ratio of the variance to the average. We denote the number of gene products by *n* (the number of proteins or mRNAs) and the Fano factor is 
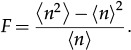


The Fano factor provides a measure to compare a probability distribution with the Poissonian distribution. The Poissonian distribution has *F*=1, while *F*<1, characterizes a sub-Poissonian distribution. The super-Poissonian distribution has *F*>1. Determining the probability distribution governing the gene product number is important because it provides some hints on the regulatory strategy of the gene. For example, a constitutive gene has *n*, which is governed by a Poissonian distribution; a sub-Poissonian distribution governs the expression of a self-repressing gene 1, while super-Poissonian distributions might indicate positive feedback (governed by a bimodal distribution) or bursty expression of a two or more genes (governed by a gamma or a negative binomial distribution).

**Figure 1:**
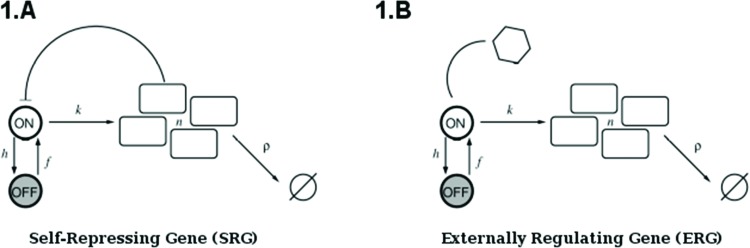
(**A**) is a representation of a self-repressing gene, and (**B**) represents an externally regulated gene. The protein number is denoted by *n,* while its synthesis (degradation) rate is denoted by *k*(*ρ*). The ON to OFF and the OFF to ON state switching rates are indicated by *h*, and *f*, respectively.

The above analysis is completed for regulation of gene expression modeled by a binary promoter assuming states ON or OFF. When the promoter is ON, there is synthesis of gene products at rate k, while no synthesis occurs in the OFF state. The gene products decay at a rate *ρ*. The rate of promoter switching from the OFF to ON state is denoted by *f* while the opposite transition occurs at rate *h*_1_ (for the self-repressing gene) or rate *h*_2_ (for the externally regulating gene). Figure 1A and 1B presents a cartoon of our simplified model for regulation of gene expression.

The scheme presented in Figure 1A and 1B corresponds to the set of effective chemical reactions presented below. The left-hand equations correspond to the self-repressing gene (SRG), while the equations for the externally regulated gene (ERG) are presented on the right. For the SRG, we denote a protein by *P*. The regulatory region of the gene is denoted by *R* and the gene state is determined by the binding of *P* to the regulatory region. The regulatory protein of the ERG is denoted by *P_e_*, and its product is *M*. The symbols on top of the arrows indicate the reaction rates.

**Table:** 

Self-repressing gene	Externally regulating gene
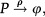	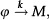
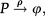	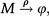
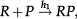	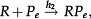
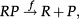	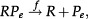

Equations 2 and 6 indicate the protein synthesis, while degradation is indicated by Equations 3 and 7. The gene switching from the ON to OFF state is indicated by Equations 4 and 8, while the opposite transition is presented by Equations 5 and 9. The system of effective reactions presented here is very simplified compared with the complexity of gene regulation and gene expression in mammals. However, such a simplification is necessary for establishing a quantitative description based on exactly solvable models.

The probability of finding the gene in the ON (or OFF) state when there are *n* gene products inside the cell is denoted by *α*_n_ (or *β*_n_). Hence, the state of the system is determined by two random variables (*m,n*), with *m* ε {*ON,OFF*} and *n* being a non-negative integer. These probabilities can be computed for a specific stochastic process governing their evolution. Here, we use a continuous-time Markov process, also known as a master equation, which is characterized by a combination of the individual transitions of the state of the system. The left-hand side of a master equation has the rate of change of the probability for the system being in a given state, while the right-hand side has the processes that cause the changes in probabilities. A positive term on the right-hand side of the master equation is a transition that brings the system to the current state, while transitions taking the system from the current state are negative.

The master equations governing the dynamics of the probabilities (*α_n_*,*β_n_*) are written below. We interpret the first term on the right-hand side and the remaining terms following the same framework. The term proportional to *k* has a positive and a negative component, *α_n_*_-1_ and *α_n_*. The former indicates that if the state of the system is (ON, *n* - 1) and there is synthesis of a gene product, the system reaches state (ON, *n*), while the second indicates that synthesis takes the system from the current state (ON, *n*) towards state (ON, *n* + 1). The master equations are written as 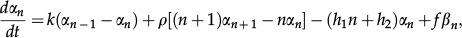


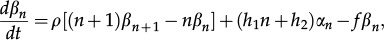


where the self-repressing gene is modeled considering *h*_1_≠0 and *h*_2_=0 because the switching rate from the ON to OFF state depends on *n*. The contrary condition, *h*_1_=0 and *h*_2_≠0, results in a model for the externally regulated gene. The solutions to Equations 10 and 11 have been obtained exactly for the self-repressing gene 38,39 and the externally regulated gene 40,41.

Living organisms have the striking capability to regulate the expression of their genes with proper spatio-temporal precision. Hence, although random variations in gene product levels are observed, these fluctuations are regulated to lie within specific ranges in normally behaving biological systems. An important issue is to find regulatory strategies underlying this precision to classify the biological functions of gene regulatory strategies. For example, it was experimentally demonstrated that self-repression reduces random fluctuations in gene expression 21,22,24, a fact also discussed under a theoretical perspective 23. However, the mechanisms enabling this noise reduction were not clear. This process is shown by writing the Fano factor as 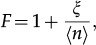


where *ξ* is the covariance between the variables (*m,n*) when the values (ON, OFF) of *m* are represented by the synthesis rates (*k/ρ*,0) to enable computation of *ξ*. The model for the self-repressing gene has a domain of regimes with *ξ*<0 generating sub-Fano probability distributions 1. These regimes occur when the gene switching between the ON and OFF states is the most frequent process compared with synthesis or degradation of gene products during a given time interval.

**Figure 2:**
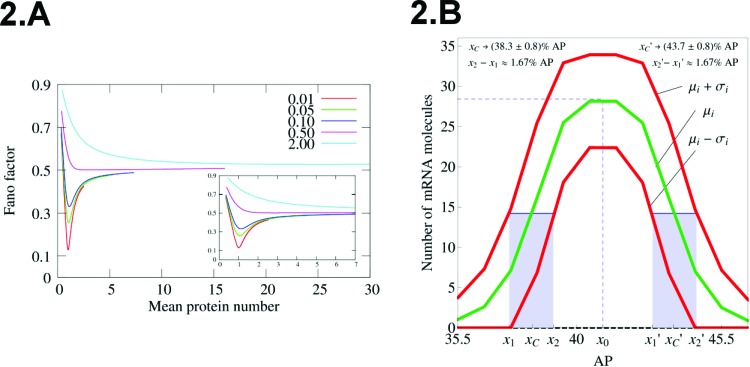
(**A**) Fano factor versus average protein number for the self-repressing gene. The value of **a** is fixed as 500. The values of b^s^ are indicated within the graph, while we varied the value of z_0_. (B) Spatial profile of mRNA average amounts along the AP axis of a *D. melanogaster* embryo. We also included the fluctuation in the positions of the borders of the peak expression along with the standard deviation of n at each nucleus along the AP axis. The positions of the borders are computed at the point where <n> is half of its maximal value at the position 41.5% of the embryo length.

Figure 2A shows the Fano factor for a self-repressing gene in the sub-Fano regime. Note the possibility of finding arbitrarily low values for *F* when <n>=1. This situation corresponds to a kinetic model in which the regulatory protein has a high affinity to the regulatory region controlling the expression of the gene. In this regime, once a regulatory protein is released from the DNA and the gene turns ON, an available protein rapidly binds to the DNA and the gene switches back to the OFF state.

The cartoon that we generated for gene regulation may appear to be a strong simplification of the whole picture in metazoans. However, we may use this approach for description of eukaryotes under specific assumptions. For example, during its early developmental stages, *D. melanogaster* embryos are characterized by a syncytium in which the cells only have their nuclei. This fact enables us to apply the gene transcription model for an externally regulating gene and use it as a first step to understand how fluctuations in mRNA synthesis relates with noise in mRNA borders' domain positioning during pattern formation.

Indeed, we carried out this approach to model the transcription of the even-skipped (*eve*) gene, which is important for the formation of a spatial pattern of protein concentration along the AP axis of the embryo that will determine specific functional segments in the adult organism 2. The *eve* mRNA spatial pattern is characterized by a Gaussian profile at the onset of gastrulation (Figure 2B). To apply our model, we assumed a one-dimensional lattice where each node has a single copy of *eve*. The lattice represents the AP axis of the embryo, and theoretical values for <n> at each node of the lattice were compared with observed values for the *eve* mRNA fluorescence obtained experimentally 42. At this stage, the challenge was to propose a method to convert the intensity of immunofluorescence into the number of mRNAs. Then, we compared the two spatial patterns at the onset of gastrulation (theoretical and experimental) and obtained a good agreement. The second stage was to compute the values of <n>±σ along the whole lattice, where σ indicates the standard deviation on *n*. Then, we compared the position of the borders of the domains and their fluctuations with the experimental data 27. The results showed theoretical fluctuations with the same magnitude as those observed experimentally. This finding was unexpected as it indicates that the required spatial precision for pattern formation in embryos can be achieved without the most precise gene regulatory strategy.

### Cell level models

**Figure 3:**
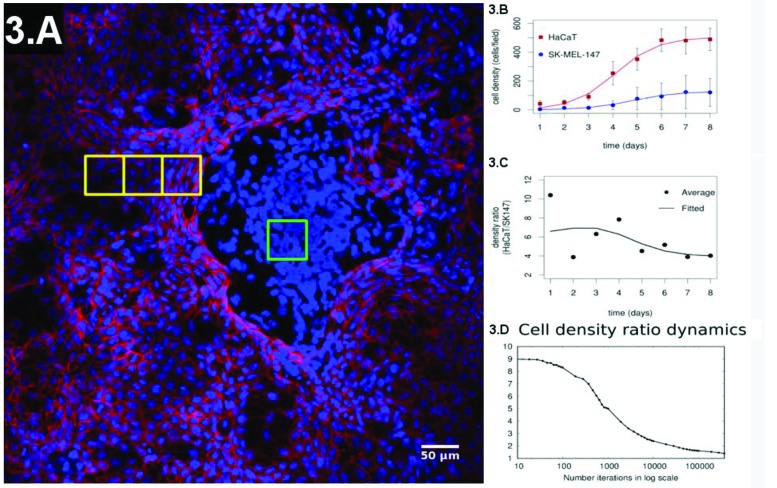
(**A**) Representative configuration of the co-culture experiment at the confluence regime. (**B**) Evolution of the individual populations until confluence is reached and their fitting by a sigmoidal curve. (**C**) Experimental ratio of melanoma to normal cells over time. (**D**) Simulation of the ratio of melanoma to normal cells over time.

There is strong experimental evidence indicating that loss of contact inhibition is a key process that enables tumors to grow and allows their occurrence within a given tissue (forming the carcinoma *in situ*) 33-36, while hypersensitivity to contact inhibition may prevent the presence of tumors 37. These effects indicate the necessity of a quantitative (and geometric) understanding of how contact inhibition affects proliferation dynamics and cellular spatial distribution in tissues. Such an approach will be useful for understanding cancer development and for designing techniques for early diagnosis and treatment. To approach this problem, we have proposed a co-culture experiment combining keratinocytes (HaCaT or normal) and melanoma (SK-MEL-147 or cancer) cells 3. We considered an initial configuration of 10:1 (keratinocytes: melanoma cells) and evaluated the cell density daily until confluence was reached. The initial configuration was composed by well-mixed populations of keratinocytes and melanoma cells. At confluence, we observed spatial patterns with normal cells being spread out and surrounding melanoma clusters (Figure 3A). In this experiment, the growth rates of the two subpopulations of cells were fitted by Gompertz logistic-like curves 43 (Figure 3B). Gompertz curves are characterized by a sigmoidal shape such that the population densities have exponential-like growth in the early phase. The growth rate reaches a maximal value and starts diminishing while cell population density asymptotically approaches its maximal value. The early density growth rate is proportional to a constant, which is the inverse of the cell division time during the early phase of culture experiments when the growth is still approximately exponential. The fitting has shown that the growth rate of both cell types had the same value, while the final proportions of the two populations diminished from 10:1 to ≈ 4:1 (Figure 3C shows the temporal evolution of this ratio). This result shows the limitation of Gompertz-like curves to describe experimental results when distinct cell types interact in the same environment. Specifically, these models have been developed in the context of predator-prey interactions in an environment with finite resource availability 44. We did not find any instance of a co-culture of melanoma and normal cells interacting similar to a predator-prey system in the literature. Hence, a quantitative description of our system required a different approach.

**Figure 4:**
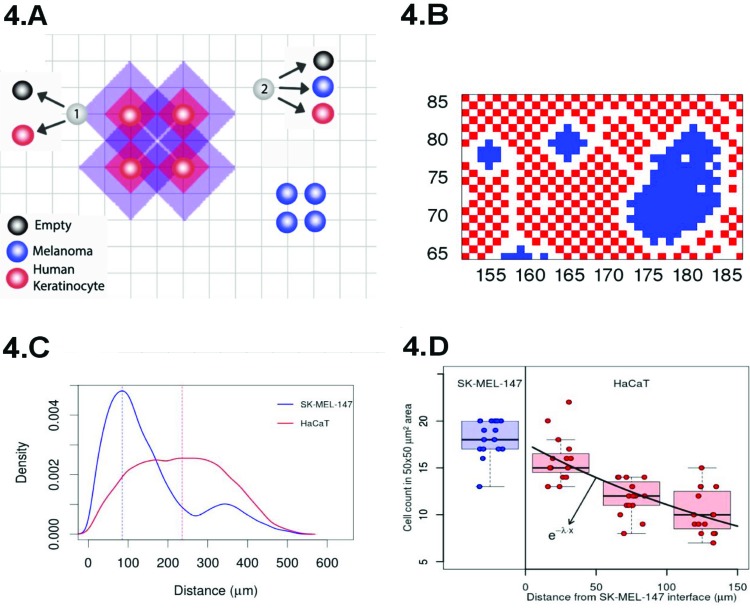
(**A**) Cartoon of our model for proliferation under different allelophylic degrees. (**B**) Spatial configuration achieved in our simulations at the co-culture confluence regime. (**C**) Experimental cell-to-cell distance distribution (the blue/red curve indicates the cancer/normal cells histogram). (**D**) Normal-to-normal (red) and melanoma-to-melanoma (blue) cell count in a 50 × 50 µm^2^ region as they are further away from the interface with the melanoma clusters.

A cartoon presenting our approach, based on the Widom-Rowlinson model 45-47, is shown in Figure 4A. The tissue is represented by a two-dimensional grid of size L×L. Melanoma (or normal) cells are indicated in blue (or red) and can occupy the grid's vertices. The distance between two cells is the smallest number of edges connecting their vertices. The latter enables us to define contact inhibition by means of an exclusion diameter around the cell represented by the shadowed areas around the red circles of Figure 4A. The vertices within purple regions cannot be occupied by melanoma cells, while the normal cells cannot occupy the vertices within the red shadowed areas. The exclusion diameters are reciprocal and show that melanoma cells can be separated by only one edge. In our model, cell type *i* (*i*=1 or *i*=2 for melanoma or normal cells, respectively) undergoes division (with rate *α_i_*), quiescence (with rate σ_i_), death (with rate *ρ_i_*) and migration (with rate *δ_i_*).

In reference 3 the dynamics of the model is established by the following Markov chain Monte Carlo method described below. A vertex *x* of the grid is selected with probability L^-2^ and its state is verified as occupied or empty. 1) For the vertex being occupied by the *i*-th cell type: *x* remains occupied (quiescence) with probability *α_i_*/*Q*; there is a probability 
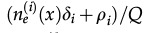
 for vertex *x* to be empty when 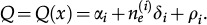. The vertex *x* becomes empty because of cell death or migration occurring with probability *ρ_i_/Q* or 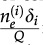. In case of migration, the cell arrives at any vertex at distance *D(i,j)* that satisfies the admissibility rule. The number of vertices around *x* that can receive the cell is indicated by 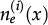 and 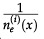 is the probability for the arrival of the migrating cell to one of those vertieces. 2) For the vertex *x* being empty: it remains empty with probability 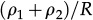 (one might define the probability using a different rate, with the cost of parameter addition); the vertex *x* may become occupied by the *i*-th cell type with probability 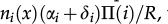, where cell division (or migration) occurs with probability 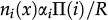 (or 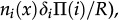, where 
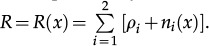
. The number of the nearest vertices around *x* allowed to receive the *i*-th cell type is denoted by *n_i_(x)*. The migrating cell around *x* is chosen with probability 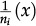 such that the original vertex becomes empty after migration. The symbol Π(*i*) is equal to 1 or 0 when the vertex *x* may or may not receive the *i*-th cell type according to the admissibility rule, and *x* remains empty for *R*=*ρ_1_*+*ρ_2_*.

Figure 3D shows the ratio of the density of normal to tumor cells of our simulations, and the curve has the same shape as the one obtained experimentally. Furthermore, in our simulations, the cell densities of the two subpopulations follow the same pattern observed in the experiments (Figure 3). Hence, it is fair to conclude that in our model, the curve has the same shape as the one obtained experimentally and is a strong candidate to quantify how different degrees of contact inhibition affect the dynamics of cell proliferation in co-culture experiments and, hopefully, in tissues.

Indeed, Figure 4B shows the spatial configuration that we obtained after simulating our model. The blue cells form clusters that are surrounded by the red ones, a pattern similar to that observed in our co-culture experiments (Figure 3A). The distribution of cell to cell distances observed in our experiment is shown in Figure 4D, and at confluence, the typical distance separating normal cells is greater than that separating cancer cells by a factor of ∼2 (see Figure 4C). This observation reinforces our geometrical interpretation of contact inhibition as an exclusion diameter and the cancer cells as allelophilic (*allelo*, the other; *phylia*, affinity). In our study, we also found the correspondence between the spatial scales of the pattern observed in our simulations and the experiments, demonstrating the agreement between the melanoma cluster distribution of perimeter ratios of the major to minor axis, areas and convex hull 3.

### Perspectives

Our results provide some perspectives for further research, and a non-exhaustive set of possibilities is discussed below. The use of a stochastic binary model for gene expression in the eve stripe 2 along the AP axis of the fruit fly embryo is needed to elucidate the effects of chemical reaction fluctuations on the spatial organization of the cells. Furthermore, the model for a self-repressing gene can be used in the context of cancer to investigate the behavior of BACH1 production under influence of a bio-metallic compound such as heme 48. BACH1 is a self-repressing transcription factor that is 48 overexpressed in triple-negative breast cancer cells. Its role as a metastasis promoter has been demonstrated, and a model for regulation of BACH1 expression level might be relevant to the development of new therapeutic approaches. Heme accelerates BACH1 decay, and we can use the self-repressing model to develop a strategy that reduces both the expected amounts of BACH1 within the cells and their fluctuations to increase cancer treatment effectiveness. Under a more theoretical perspective, we may also consider investigating the meaning of the symmetries of the stochastic binary models 23,26 aiming to model two interacting genes.

For the cell level models, one may propose a Markov chain to approach tumor cell phenotypic heterogeneity. Tumor progression can yield changes in its architecture that result in tumor cell death or the development of invasive phenotypes because of the scarcity of space and resources 49-52. Additionally, environmental cues may modulate the expression of transcription factors regulating the internal cell dynamics 53-56. Consequently, at a random time, a cell may have its phenotype transformed from a predominantly proliferative one to an invasive one. This phenomenon suggests proposing a cell level model for tumor progression based on two phenotypes, where the invasive phenotype originates from the proliferative one. The dynamics is simulated by a Markov chain with transition rates dependent on the population size as an effective representation of homeostatic mechanisms. Alternatively, the use of the stochastic model for contact inhibition may also be extended to the condition in which there are three or more interacting cell types. In that case, we may start with three cell states, accounting for keratinocytes, melanocytes and melanomas. Here, the melanomas would result from a modification of melanocytes, and we may use these results to investigate the conditions for the progression of a melanoma *in situ* from a normal condition. Reference 3 presents simulations in a 2-dimensional grid to describe the results obtained with culture or co-culture experiments. A next step is to construct three-dimensional grids to enable us to describe *in vivo* experiments and, hence, obtain a richer picture of carcinogenesis. One natural challenge of such an approach is to establish the grids' topology with different numbers of nearest neighbors. These simulations will enable us to develop new imaging analysis tools that will be useful for a quantitative spatial characterization at different stages of carcinogenesis. These data may lead to the development of non-supervised tools for tissue characterization.

Figure 3A shows four yellow squares, one within a melanoma cluster and the remaining three within the normal cells in a sequence starting at the interface between the two domains. Inspection is sufficient to verify that normal cells near melanomas are closer to each other than those that are further from the cluster. The cell density decreases exponentially, suggesting the existence of a molecular mechanism dependent on the presence of the melanoma cells to change the cell's exclusion diameter. This finding indicates the necessity of combining the approaches for the cellular and molecular levels for a better understanding of cancer biology. It will be useful to determine how molecular level fluctuations originate cancer heterogeneity.

Our investigations on the molecular mechanisms of carcinogenesis may also have implications for the analysis of random effects of low-dose and low-dose rates of ionizing radiation 57,58. Radiation therapy is estimated to account for 50% of cancer treatment cases, and this treatment may play a role in the late appearance of tumors. Hence, understanding how low-dose and low-dose rates of ionizing radiation affect cells is an important scientific problem with clinical implications. For these regimes, one expects the effects of ionizing radiation to be stochastic such that it is natural to employ an approach based on probabilistic theory. Initial attempts at stochastic modeling of biological effects are based on target theory 59, while more detailed deterministic models have been proposed recently to account for DNA repair mechanisms of mammalian cells 60. For the latter, we will employ the Langevin technique to evaluate randomness in deterministic models. In a different research direction, one may notice that 90% of radiation treatments use radiofrequency-driven linear accelerators of electrons (RF-Linac). These RF sources are not very precise and may lead to radiation exposure of both the tumor and neighboring cells. Hence, continuous efforts have been made to identify new radiation sources, including laser-accelerated electrons for the generation of tunable and quasi-energetic X-ray sources 61,62. The development of this technology relies heavily on computational simulations of the laser plasma interactions devoted to the optimization of the X-rays generated through electron acceleration by lasers. These simulations may guide experimental designs for generation of X-rays for clinical purposes.

## AUTHOR CONTRIBUTIONS

Sabino AU, Vasconcelos MF, Sittoni MY, Lautenschlager WW, Queiroga AS and Morais MC collected the data, wrote the manuscript and approved the final version of the manuscript to be published. Ramos AF designed the study, collected data, interpreted the results, wrote the manuscript and approved the final version of the manuscript to be published.
